# Cancer suspicion in general practice, urgent referral, and time to diagnosis: a population-based GP survey nested within a feasibility study using information technology to flag-up patients with symptoms of colorectal cancer

**DOI:** 10.3399/bjgpopen17X101109

**Published:** 2017-10-04

**Authors:** Elaine Kidney, Sheila Greenfield, Lindy Berkman, George Dowswell, William Hamilton, Sally Wood, Tom Marshall

**Affiliations:** 1 Research Fellow, Institute of Applied Health Research, University of Birmingham, Birmingham, UK; 2 Professor of Medical Sociology, Institute of Applied Health Research, University of Birmingham, Birmingham, UK; 3 Patient Representative, Institute of Applied Health Research, University of Birmingham, Birmingham, UK; 4 Senior Research Fellow, Institute of Applied Health Research, University of Birmingham, Birmingham, UK; 5 Professor of Primary Care Diagnostics, Medical School, University of Exeter, Exeter, UK; 6 Clinical Research Fellow, Institute of Applied Health Research, University of Birmingham, Birmingham, UK; 7 Professor of Public Health and Primary Care, Institute of Applied Health Research, University of Birmingham, Birmingham, UK

**Keywords:** colorectal cancer, primary care, diagnosis, referral, barriers, over-referral

## Abstract

**Background:**

Patients with symptoms of possible colorectal cancer are not always referred for investigation.

**Aim:**

To ascertain barriers and facilitators to GP referral of patients meeting the National Institute for Health and Care Excellence (NICE) guidelines for urgent referral for suspected colorectal cancer.

**Design & setting:**

Qualitative study in the context of a feasibility study using information technology in GP practices to flag-up patients meeting urgent referral criteria for colorectal cancer.

**Method:**

Semi-structured interview with 18 GPs and 12 practice managers, focusing on early detection of colorectal cancer, issues in the use of information technology to identify patients and GP referral of these patients for further investigation were audiotaped, transcribed verbatim, and analysed according to emergent themes.

**Results:**

There were two main themes: wide variation in willingness to refer and uncertainty about whether to refer; and barriers to referral. Three key messages emerged: there was a desire to avoid over-referral, lack of knowledge of guidelines, and the use of individually-derived decision rules for further investigation or referral of symptoms. Some GPs were unaware that iron deficiency anaemia or persistent diarrhoea are urgent referral criteria. Alternatives to urgent referral included undertaking no investigations, trials of iron therapy, use of faecal occult blood tests (FOBt) and non-urgent referral. In minority ethnic groups (South Asians) anaemia was often accepted as normal.

Concerns about over-referral were linked to financial pressures and perceived criticism by healthcare commissioners, and a reluctance to scare patients by discussing suspected cancer.

**Conclusion:**

GPs’ lack of awareness of referral guidelines and concerns about over-referral are barriers to early diagnosis of colorectal cancer.

## How this fits in

UK mortality rates from colorectal cancer remain higher than in comparable countries and patients with symptoms are not always referred for investigation. This study found GPs who were reluctant to refer patients who met urgent referral criteria. Their reasons included poor knowledge of urgent referral criteria, lack of suspicion of common symptoms such as iron deficiency anaemia, pressure to reduce referrals, and a desire to avoid scaring patients. This reluctance to refer hinders early detection.

## Introduction

In the UK, survival after diagnosis of colorectal cancer has been observed to be poorer than in other countries, partly due to diagnosis and treatment at a later stage.^1–4^ For one-quarter of patients in 2007–2008, time between meeting NICE 2005 referral criteria^5^ and diagnosis was over 6 months.^6,7^ Annual incidence of colorectal cancer is 66 per 100 000 population, so a full-time GP will expect to see only one case per year.^8,9^ This relative unfamiliarity, coupled with the many possible symptoms makes it difficult to decide which patients to refer for diagnostic investigation. The CREDIBLE study investigated the feasibility of using electronic patient records to flag up patients aged 60–79 years with symptoms meeting NICE urgent referral criteria for investigation of suspected colorectal cancer. These can be thought of as red flag symptoms or warning signs. They include iron deficiency anaemia, persistent diarrhoea, rectal bleeding (NICE 2005 urgent referral criteria for colorectal cancer)^5^ or positive FOBt. The previously published quantitative outcomes of the CREDIBLE study^10^ found considerable variation in diagnostic and referral action undertaken by GPs for patients flagged up as meeting contemporary NICE urgent referral criteria. Many patients with uninvestigated red flag symptoms were considered by their doctor as not needing further investigation. As part of the CREDIBLE study^10 ^the authors also undertook qualitative research exploring attitudinal and contextual influences on investigation and referral of patients to secondary care through interviews with general practice staff. This study, which focuses on individual GP-level or GP practice-level barriers to GP referral of patients specifically meeting NICE urgent referral criteria for colorectal cancer, is one of the few to examine barriers to referring patients for suspected colorectal cancer.

## Method

### Participant selection and characteristics

The lead GPs and practice managers in all 22 practices participating in the CREDIBLE study^10^ were invited for interview. It was important to conduct a joint interview to develop a deeper understanding of barriers and facilitators at both the individual GP level and the practice level. The practice manager provided context from the perspective of practice organisation.

### Interviews

Data were collected between January and June 2014, towards the end of the study, through face-to-face semi-structured interviews lasting a median of 39 minutes (range 25–﻿66 minutes). Participants received an information sheet ahead of interview, provided informed consent and the interview was recorded on audiotape.

Interviews took place at the GP surgery, apart from one at the University of Birmingham, and were conducted by two non-clinical health researchers. One was an experienced qualitative researcher and the other had more in-depth knowledge of the practices resulting from working on the CREDIBLE study. One interview was carried out by one researcher alone. The original topic guide focused on views about the use of software to flag up patients meeting urgent referral criteria for colorectal cancer. However in light of varied responses to dealing with flagged up patients, interview schedules were adapted iteratively^11^ to explore reasons for not referring patients with documented referral symptoms, or for relying on FOBts (Box 1, Section 3). One researcher led the interview, while the other focused on section 3 of the interview schedule after auditing which symptoms had been followed up in practice. Recorded interviews were transcribed verbatim, and checked for accuracy at least twice. One interviewee gave permission for written notes but not audiotaping. In this case, the researchers independently wrote up the interview and compared notes for accuracy.

### Analysis

The data were analysed thematically, searching for GP text relating to referral and investigation at GP, patient, practice, secondary care, and NHS systems level.^12^ Frequent triangulation within the multidisciplinary team ensured thorough and consistent coding. This study presents data relating to investigation and referral solely from the GP perspective.

## Results

Eighteen of the 22 practices participated and 18 interviews were conducted with 18 GPs, including partners, senior partners, salaried GPs, and locums, and 12 non-clinical staff (11 practice managers and one information technology manager) across those 18 general practices. No-one was available for interview in four practices: two declined, one was unable to participate because of time commitments, and in one both the GP and practice manager involved in the original study had left.

Participating practices served predominantly deprived areas in the urban West Midlands of England, with registered populations between 2000 and 27 000 patients, some with high proportions from minority ethnic groups. Organisational structures varied from single-handed to partnership-held to nurse/practice manager consortia. Both teaching and non-teaching practices participated. Table 1 shows the practice characteristics, including those who did not participate in interviews.

**Table 1.** Participant and practice characteristics

Interview number	Interviewees	Practice size (number of patients)
1	GP1	4000–8000
2	GP2, PM1	4000–8000
3	GP3, PM3	<4000
4	PM4	<4000
5	GP4, GP5, PM5, IT1	4000–8000
6	GP6, PM5	8000–12 000
7	GP7	8000–12 000
8	GP8	<4000
9	GP9, GP10, PM6	4000–8000
10	GP11	>12 000
11	GP12, PM7	4000–8000
12	GP13, PM8	4000–8000
13	GP14	>12 000
14	PM8	<4000
15	GP15, PM9	4000–8000
16	GP16	<4000
17	GP17	<4000
18	GP18, PM10	4000–8000
19	Unavailable for interview	>12 000
20	Unavailable for interview	<4000
21	Unavailable for interview	<4000
22	Unavailable for interview	<4000

IT = IT manager. PM = practice manager.

This study presents evidence on barriers to referral at individual GP and practice levels. A wide variation in referral of symptoms and uncertainty about whether to refer was found. Initial coding revealed that referral and investigation appeared to be greatly influenced by the GPs’ beliefs and attitudes about which symptoms should be referred. Two major themes were identified: variation in referral of symptoms and uncertainty about whether to refer; and barriers to referral. Each of these had a range of sub-categories which are discussed below. 

### Variation in referral of symptoms and uncertainty whether to refer

While some GPs closely followed guidelines^^5,13^^ there were examples of deviations for almost every referral criterion (Box 2). Descriptions of clinical practice were often prefaced by 'we' indicating that other colleagues followed the same practice. When given examples of non-referral found in practice, GPs elaborated on personal decision-making processes, including detailed history taking and looking for additional evidence before referring; for example, history, examination, or investigations that would raise their suspicion of cancer.

#### GP suspicion

It was clear that all GPs would refer patients if they were suspicious of cancer, but not all symptoms flagged up as part of the CREDIBLE project (which identified patients with symptoms meeting NICE urgent referral criteria) were viewed as suspicious:

*'I don’t think it's* [the study] *altered practice. I think it's kept some of the not so hard red flag symptoms in people's minds, so just thinking about …' *(GP11)

With strong suspicions, all GPs said they would refer via the 2-week wait pathway or more quickly and on three occasions, same-day referral by GPs was mentioned.

#### Anaemia

Although iron deficiency anaemia in an adult aged >60 years is one of the criteria for urgent referral, it was not universally perceived as a strong indication for referral and in the absence of additional symptoms such as rectal bleeding, bowel problems, or weight loss, colorectal cancer was often not considered (Box 2 quote 1). Four general practices cited the use of FOBt to help decide whether to refer, while others looked for weight loss in addition to anaemia. Some GPs would prescribe iron supplements for anaemic older patients and monitor symptoms.

#### Persistent diarrhoea

Some GPs indicated that referral for persistent diarrhoea was more likely when accompanied by additional symptoms such as weight loss (Box 2 quote 2).

#### Rectal examination

There was variation in carrying out rectal examinations, with mention of colorectal cancer diagnosed in patients with rectal bleeding attributed to haemorrhoids (Box 2 quote 4).

### Barriers to referral

Four key barriers to referral were identified by GPs.

#### Poor knowledge of NICE referral criteria and pathways

In some cases, non-referral of patients with symptoms was explained by GPs’ own poor knowledge of colorectal referral guidelines or the perceived poor knowledge of others:

*'I'll have to look at it and familiarise … Persistent diarrhoea what does persistent* [mean]*, you know, how long?'* (GP10)

*'I think sometimes doctors aren't sensitive to the fact that if somebody presents with a bit of blood in their diarrhoea and didn’t see its inference.'* (GP15)

*'Probably even the colorectal problem we're not too brilliant on this particular subject. We are still learning.'* (GP18)

There was also uncertainty about whether or not to use the '2-week wait' pathway, whether to refer directly to colorectal surgeons or to gastroenterology and, in the case of anaemia, whether to refer first for lower or upper gastrointestinal investigation.

#### Resource constraints and professional norms ('the policeman inside your head')

There was frequent mention that patients should not be referred unnecessarily, for the good of the wider NHS. It was found that resource pressure, perceived to come from the clinical commissioning groups (CCGs) made some doctors think twice about referring: 

*'They* [the CCG] *say, you know, budgets are very stretched and people are referring too much, referral rates are high.'* (GP1)

* 'GPs are encouraged not to send patients to the hospital ... if I referred all these patients with diarrhoea or anything, they’d say* [the CCG would say] *that I referred 1500 patients with all these problems and none of them have been diagnosed with bowel cancer. So they’d say my referral rate was poor.'* (GP2)

Although cancer referrals were not targeted by the CCG for reduction, the message of reducing 'unnecessary' referrals was often in the back of GPs’ minds: 

*'If it's costing us more, irrespective of every other thing, we don't do it. The emphasis has moved from benefits and quality of care more towards is it within our financial means.'* (GP15)

This appeared to be reinforced by peer pressure: general practices aimed to be at or below median CCG referral rates in referral league table:

*'Nobody cares if you’re not referring enough. Not referring gives like a safe shield for the practice.'* (GP2)

Three doctors challenged the pressure not to refer, GP15 described how budgets were balanced by use of good history taking and in-house investigations and two GPs advocated referring more patients to rule out cancer (GP14) or to be sure of not missing cancers:

*'You sometimes worry more about the ones* [doctors] *who just refer the exact cancer cases, because you think you really ought to be referring more non-cancer cases in order to be sure that you're picking up all the cancers.'* (GP7)

#### Reluctance to refer: not wanting to scare the patient

Pressure from patients may make the GP more likely to refer:

*'I think the most dramatic things in the mind of the patient and the relatives, is not finding the cancer in good time.'* (GP15)

There was also evidence of GPs holding back from discussing cancer and avoiding referral in order to prevent patients from becoming scared:

*'So I don’t know how far I go or how much I instil in them and say, "Look, this could be that you may be suffering from cancer" and things like that. You don’t want to scare the patient away. I’d be referring a lot of patients unnecessarily and building up their anxiety as well. Because everybody worries about cancer, especially bowel cancer because it’s a nasty sort of a thing, so you don’t want to be referring unnecessarily.'* (GP13)

Patients identified as already being anxious might be less likely to be referred:

*'I know which patients, where the anxiety level would be with which patient. With some patients I could say anything and explain to them my logic on things. Sometimes I don’t say/do anything until I get something positive and then I’ll mention it, because I know that the next referral will be to a psychiatrist if I mention the word "C" because they’ll have a mental breakdown.'* (GP13)

#### Acceptance and normalisation of mild anaemia

Anaemia was common and there was evidence of acceptance of mild anaemia and a tendency to manage anaemia in primary care:

*'We're finding that this is a really huge burden … the number of patients we have in their 70s, 80s, 90s, who've been on medication for a long time, they have a little bit of a borderline anaemia and then we … we often wonder well, should this now go to a 2-week *[wait] *or should we do a few investigations beforehand. So this is a real issue, actually.'* (GP10)

*'Because anaemia itself has differential diagnosis. Everything causes anaemia. So we need to go for a good history, which will give you almost 50% success in what you are thinking about. Then do a proper investigation.'* (GP18)

*'The symptoms could be very weak and you can’t just,* [say] *any uncertainty and then you refer, you just have to try and see ... maybe go into the symptoms a little bit more and … if it’s not suspicious otherwise, do some investigations, such as an PSA* [prostate specific antigen test]* or BCSP* [bowel cancer screening programme] *with a full blood count. Then, if there isn’t anything, try and treat the symptoms for a short while and see what happens, and then refer them.'* (GP 13)

There was a reluctance to refer for urgent colorectal cancer investigation. Some GPs mentioned that anaemia can have a number of causes and many of these are more common than colorectal cancer:

*'I suppose part of the problem certainly locally here is that there is a high incidence of dietary iron deficiency and also due to women who are reproductive age tend to develop that sort of side of things so that may desensitise some GPs to actually asking the question, what's the diagnosis here, before starting treatment.'* (GP7)

GPs gave normalising explanations for mild anaemia in South Asian patients, attributing this to lack of dietary iron:

*'I don’t know whether this has been picked up or not, but some of the time it* [iron deficiency anaemia] *could be dietary as well. People’s diets are not very brilliant. I’ve just had a patient today who said, "Can I have some vitamins?" I said, "Why? Don’t you eat a lot of fruit and everything?" He said, "No, we don’t eat any fruit." I thought then that it can’t be helped.'* (GP13; practice with a high proportion of Asian patients)

*'We do know that the diet is the most common cause of iron deficiency anaemia, because many communities don’t eat meat, they don’t eat certain foods that are highly rich in iron. With the experience of the GP in that area, sometimes we just give dietary advice and don’t give iron tablets. We’ll do the blood test and that’ll be fine for the cause of … That’s one particular area where the GPs will be more reluctant to do a referral. They’ll know the family, and that the father is anaemic, the mother is anaemic, and the children are anaemic, they share the same diet.'* (GP2; practice in which Asian Indian was the largest ethnic group)

### Diagnostic strategies found to improve or hinder early detection of colorectal cancer

Early referral was favoured by following NICE guidelines and by having a clear protocol for referring anaemic patients if their ferritin levels were low:

*'I'm very protocol driven … I said, "Anyone that's got iron deficiency anaemia will mean they automatically get referral to this. If their serum ferritin is low they automatically get referred".'* (GP 17)

## Discussion

### Summary

In the CREDIBLE study software was used to search electronic patient records to generate a list of patients aged 60–79 years meeting contemporary NICE urgent referral criteria: persistent diarrhoea, iron deficiency anaemia, rectal bleeding, or positive FOBt.^10^ These data indicate that GPs in the current study were often reluctant to refer these patients for a variety of reasons. They were aware of pressure to avoid over-referral and wished to avoid being identified as having a high referral rate. Some GPs considered that referral would scare patients and weighed this against a low perceived risk of cancer. Before referring, some GPs therefore required additional evidence: their own suspicion of cancer, or clinical features such as weight loss, positive FOBt results, or bowel symptoms. NICE red flag symptoms alone were not regarded as sufficient. Anaemia was often perceived as common and therefore not always warranting investigation. Because of lack of familiarity with referral guidelines, some GPs used personally devised decision rules and diagnostic strategies which hindered further investigation. 

From these data it appeared that two groups of patients may be at greater risk of non-referral: minority ethnic groups with anaemia, because this is attributed to diet, and patients with anxiety issues, which could be exacerbated.

### Strengths and limitations

The interval between the onset of symptoms and eventual diagnosis is affected by the patient, GP, secondary care clinician, and the healthcare system.^14^ This study focused only on the interval between first presentation to primary care and first referral to secondary care.

Interviews may be subject to social desirability bias,^15^ but GPs were confident in describing referral practice which went against NICE guidelines. Interview findings were corroborated by triangulation with data from the same general practices showing non-referral and long diagnostic delays in patients with anaemia and diarrhoea.^10^ These findings are also consistent with the shorter diagnostic intervals seen in patients presenting with rectal bleeding.^16^ The 18 GP responders were all from urban general practices serving deprived, multiethnic populations in one region of England. It is not possible to comment on whether we would obtain similar findings from practices serving more affluent populations in other regions. However participating general practices were not homogenous and varied in size and organisational structure. Only half the interviews included both practice managers and GPs but findings were similar from individual and joint interviews. Although this study focused on GP perspectives further analysis of joint interviews with practice managers will be undertaken to provide a broader practice organisational perspective.

These findings are based on interviews conducted in 2014 and referral guidelines have subsequently been updated. However some of the findings are consistent with similar observations many years previously which suggests they may still be applicable today. For example, these findings in relation to lack of investigation of anaemia hav been observed for at least 2 decades.^17,18^

### Comparison with existing literature

This study is one of very few to investigate GPs’ reasons for not referring patients meeting urgent referral criteria. Others have observed that GPs do not always refer patients for investigation of suspected cancer, use individually devised decision rules in relation to FOBt and express concerns about over-referral.^19–22^ GP concern about cancer referral causing anxiety is supported by evidence: investigation of suspected cancer affects quality of life.^23^ GPs may fail to provide explanations to patients referred for investigation of suspected cancer, which may be to avoid frightening them.^24^ Nevertheless there is clear evidence that four-fifths of patients prefer to be investigated even if the risk of cancer is 1% (consistent with persistent diarrhoea).^25^ Higher use of urgent referral pathways may reduce cancer mortality^26^ and NICE recommends urgent referral for anaemia and persistent diarrhoea in the over 60’s at low levels of risk.^27^

Common GP strategies to refine the diagnosis may cause delays. Heneghan *et al* ^^28^^described a three-stage model of diagnosis in primary care:

1. initiation of diagnostic hypotheses;
2. refinement of the diagnostic hypotheses; and
3. defining the final diagnosis, with various strategies used at each stage.

In the context of Heneghan’s model the current findings suggest that early referral was favoured by following NICE guidelines and by having a clear protocol for referring anaemic patients if their ferritin levels were low (further information available from the authors on request). Although more recent guidelines suggest that weight loss is a useful symptom,^27^ diagnosis by pattern recognition^28^ may result in delay until later symptoms present themselves. While GP suspicion is a good indicator of serious disease, absence of suspicion does not rule it out.^29,30^ Further investigations in primary care may not help: FOBt tests in symptomatic patients are associated with frequent false negatives.^27^

International research suggests these findings may have wider significance. Others have reported variations in individual GPs’ tendency to refer patients and that patients with anaemia are not investigated. A video-vignettes study of Australian GPs found one in eight cases of patients with cancer symptoms would not be referred.^31^ In a survey of GPs in Denmark, GPs suspected cancer in less than half of all actual cancers and diagnostic delays increased by a month if the GP considered symptoms to be 'vague' rather than 'alarm'.^32^ Anaemia is the most common missed opportunity for colorectal cancer diagnosis in a number of countries.^33,34^ Furthermore, low referral rates have been linked to cancer survival internationally. An online survey of primary care providers from 11 jurisdictions in six countries found a positive correlation between readiness to investigate and cancer survival rates.^35^ 

This study took place 9 years after publication of the NICE (2005) cancer referral guidelines, when they might be expected to be reasonably well accepted and followed. Concerted efforts have been made in the UK in recent years to increase detection rates of cancer, including national FOBt bowel cancer screening, urgent referral (2-week wait) pathways, and the development of clinical decision aids for colorectal cancer referral^36,37^ but under-referral still exists. Meanwhile, the NHS faces increasing budgetary pressures, directed at GP CCGs.^38^ New NICE guidelines encourage GPs to use their judgement when assessing risk of cancer, with much lower threshold levels^^27^^ but these findings suggest this may be ineffective if the GP’s assessment of risk is lower than the actual risk or if the GP is deterred by pressure to reduce spending

### Implications for research and practice

Clinical guidelines intend patients meeting urgent referral criteria for colorectal cancer should be referred promptly. By lowering the risk threshold, new guidelines aim to increase urgent referrals.^^27^^ Lower gastrointestinal endoscopy, with removal of precancerous polyps, reduces mortality from colorectal cancer,^39^ with protection lasting several years.^40^ High use of urgent referral (2-week wait) pathways appears to reduce cancer mortalities.^^23^^ However GPs’ concerns about alarming patients and perceived external pressures from CCGs to reduce referrals directly undermine this strategy.

Patients identified as being at risk of cancer through FOBt screening are routinely referred, without opportunity for GP discretion. Professional autonomy allows GPs to exercise discretion in relation to referral of patients with signs and symptoms but also may conflict with the intention that patients meeting urgent referral criteria are referred. GPs who follow guidance refer earlier than those who pursue a 'personal' or more 'ad hoc' decision-making strategy. Diagnostic strategies used by GPs may hinder referral because referral for investigation of suspected cancer does not require a definitive diagnosis.

Due to concerns about over-referral and scaring patients, many GPs are reluctant to refer patients who meet urgent referral criterial for colorectal cancer. Healthcare commissioners should support and encourage GPs to refer patients meeting urgent referral criteria for cancer.

Further research could test the belief that anaemia is less predictive of colorectal cancer in minority ethnic groups. Research also is needed to identify the strategies most likely to promote referral. This could include drawing attention to the need to increase referrals, highlighting practices that refer too few patients and making GPs aware that most symptomatic patients prefer to be investigated. Particular attention may be needed on strategies to increase referral thresholds for overly-anxious patients. 

**Box 1.** Interview schedule.

**CREDIBLE study experience** Having gone through a period of us running the searches at your practice, has it changed any aspect of practice? Has it changed referral thresholds? Made you question current practice? Raised awareness of symptoms? Influenced decision making? Changed your view of decision support systems? Do you think it this something that should be done as standard practice in primary care? What the advantages of this kind of case finding? What are the disadvantages? Do the advantages outweigh the disadvantages? What do you think needs to be done to embed this into routine practice? Who would need to do what? Instead of a monthly review of patient records, would you prefer a system that flagged up patients as soon as they met referral criteria?
**CREDIBLE study context: local** Where do you put early diagnosis of cancer in your list of priorities? What about your colleagues here —﻿ do the other GPs see things differently? Does referring patients for further investigations improve or damage your relationship with them?
**CREDIBLE study context: wider** Do you agree with the NICE guidelines on referral? 2-week wait (urgent) referral for investigation of: persistent diarrhoea and/or rectal bleeding (both if aged ≥40 years, either if aged ≥60 years) unexplained iron deficiency anaemia (for investigation of both upper and lower gastrointestinal cancer) rectal mass abnormal rectal exam 'In patients with equivocal symptoms who are not unduly anxious, it is reasonable to use a period of "treat, watch and wait" as a method of management' (Weight loss and abdominal pain are risks but not NICE referral criteria.) Are there any times when you’ve been **uncertain** whether or not to refer for urgent investigation? **Anaemia** seems to be an area with wide variations in practice. What are your preferences for: diagnosis treatment referral Can you say what a proper GP should do with regard to early diagnosis of colorectal cancer? How does this CREDIBLE approach support or undermine your sense of being a proper GP? Do you ever have difficulties deciding which consultant to refer to? If you are uncertain about diagnosis, is it better to wait and see or to refer to a specialist? Have you ever been criticised by a consultant when you have made a referral? Is it better to risk annoying a specialist or risk missing a chance to diagnose earlier? Is it better to risk worrying a patient or risk missing a chance to diagnose earlier? **If a Faecal Occult Blood test (FOBt)** is negative does this influence your decision to refer? What would your reaction be if we said we’d found examples of some GPs who decide not to refer patients with symptoms because they have had a negative FOBt? Would you yourself ever decide not to refer a patient with symptoms if a FOBt was negative? (*Here reinforce why NICE recommends they should not a) not reliable enough to rule out, b) wastes time getting a FOBt done and lengthens interval time between symptoms and diagnosis*) Do you ever use online learning tools? There is a new online learning tool (with CPD accreditation): Suspected lower gastrointestinal tract cancer: when you should refer (http://learning.bmj.com/learning/search-result.html?moduleId=5003316). Would it have helped you assess the lists of our patients we’ve flagged up if we’d been able to point you in its direction before we started the study? How might we best influence other GPs to refer more appropriately?

**Box 2.** Deviations from NICE guidelines

**1. Anaemia** *1.1. 'When they have iron deficiency anaemia, initially we will be doing the ferritin test. If they’re low and straightforward we will give them treatment.' *(GP2) *1.2. 'We always take the symptoms into account, to see whether anybody has got a bowel problem to start with. If there are associated features, like weight loss or passing blood in the stools or anything, then we investigate the first line management* [in general practice]*.' *(GP2) *1.3.* **Interviewer:** *'How much time and effort do you think a good GP should spend to try and work out the diagnosis?'* **GP18: ** *' I think about two or three, maybe maximum four sessions with patient after having asked for investigation. At least two sessions.'*
**2. Diarrhoea** *2.1 'Ideally, if there’s weight loss associated, as well as chronic diarrhoea, then definitely I will be looking for a cause.'* (GP2)
**3. Rectal bleeding** *3.1 'When somebody comes with PR bleeding, we do examination of PR but our knowledge is not 100%. And we get confused sometimes whether it's some anal problems or whether it's piles. Piles usually that's detectable. And anal fissure is significant. So then we are in a bit of a dilemma because it has happened with our practice and GPs, when they referred it turned out to be anal fissure, it's not cancer, when they had the colonoscopy and all that.'* (GP18)
**4. Rectal examination** *4.1 'I have seen cases, I've got patients with rectal bleeding and they’ve been told it's piles and its stopped and they have bleeding again, and every time its piles, piles, the doctor told me that —﻿ and it turned out to be rectal carcinoma.'* (GP6) *4.2 '… And some people who are* [were] *treated as oh, it’s just piles and use the* [FOBt] *screen. Or the PR was not done.'* (GP16) *4.3 '… Or where they've got a past history of piles and I'm satisfied that it's piles where I've PR'd them previously and they bleed again. It's difficult. You can't refer and you don't refer every single one that looks that way.'* (GP17)
**5. Using an FOBt to decide** *5.1 'If there is a bowel problem, bowel irregularities, we definitely check a stool for occult blood. We definitely do that.'* (GP 18) *5.2 'Weight loss and occult blood test, if it is positive, definitely creates some kind of suspicion for us.'* (GP18)*5.3 'Quite often what we do is, when we're not sure exactly whether to refer or not, then we do an FOBt, and then of course according to the results we'll act on whatever is necessary.'* (GP10) *5.4 'If the faecal occult blood is negative but the person has lost weight or has diarrhoea, I tell the patient that they don’t have to worry. If the patient is losing weight and having chronic diarrhoea then that needs to be looked into. I would still be referring them, even if their faecal occult blood is negative.'* (GP2) 5.5. **Interviewer:** 'GPs have sometimes used FOBt as part of a preliminary process before they’ve thought about referral. We wondered if you’d seen it as part of that?' **GP13: ** *That was very good teaching, ... So if they* [patients] *had no more symptoms, then I felt reassured* [by the negative FOBt]*. If they’ve got symptoms persisting then I won’t rely on it* [FOBt]*. If there are no symptoms persisting then I would rely on it.'*
**6. Ordering further tests, sometimes doing in-house test to save money** *6.1 'If we see microcytic anaemia in particular then we will not necessarily investigate them to the extent of sending them for a sigmoidoscopy but we will actually go through iron studies and we will ensure that we do a digital rectal examination or examine their abdomen and look for a cause. If we can't* [find one]*, then we will refer.'* (GP 15) *6.2 'If you think about 10 years ago, everybody who had diarrhoea was sent to the hospital, whether they were worried about cancer or not, and they were diagnosed in the hospital. Then all this cost and everything came up.'* (GP2)
